# Assessing the Use of Twitter to Share Canadian Residency Match Information During the COVID-19 Pandemic

**DOI:** 10.7759/cureus.42548

**Published:** 2023-07-27

**Authors:** Lauren Viola, Kameela M Alibhai, Emaan Chaudry, Julia Kemzang, Karima Khamisa

**Affiliations:** 1 Schulich School of Medicine and Dentistry, Western University, London, CAN; 2 Department of Surgery, Faculty of Medicine, University of Ottawa, Ottawa, CAN; 3 Department of Medicine, Faculty of Medicine, University of Ottawa, Ottawa, CAN; 4 Division of Hematology, Department of Medicine, University of Ottawa, Ottawa, CAN

**Keywords:** twitter, social media, residency match, covid-19, carms

## Abstract

Purpose

In their final year, medical students explore prospective residency programs by completing visiting electives and attending interviews during the Canadian Resident Matching Service (CaRMS) process. Due to COVID-19, visiting electives and in-person interviews were suspended, leaving residency programs searching for alternate ways to share CaRMS information with applicants. This study evaluates the utility of Twitter to share CaRMS-related information prior to and during the pandemic.

Methods

Primary tweets published from three CaRMS cycles between 2018 and 2021 were identified using the analytics tool Vicinitas. The type, content, and language of tweets and the date and location of publication were extracted. Demographic data about tweet creators were determined using provincial regulatory college databases and institutional websites. Descriptive statistics were employed for categorical variables. All tweets were deductively analyzed.

Results

Of the 1,843 tweets, 603, 472, and 768 were published during the 2018-2019, 2019-2020, and 2020-2021 cycles, respectively. Most tweets were written in English (97.4%) and by medical students (29.5%) affiliated with Ontario universities. The most common types of tweets were supportive messages (29.1%), reflections about CaRMS (24.7%), and positive match results (20.8%). Rurally located institutions experienced the greatest increase in the total number of tweets between the pre- and full-COVID cycles.

Conclusion

Since COVID-19, Twitter has been increasingly used by medical professionals to share CaRMS-related information, primarily to promote programs and advertise CaRMS events. Given the environmental and financial benefits, CaRMS interviews will likely remain virtual, which highlights the ongoing need for residency programs to use social media platforms to share information with prospective applicants.

## Introduction

During the final year of medical school, senior medical students (SMS) must select their preferred specialties, identify schools of interest, and apply to the Canadian Resident Matching Service (CaRMS) portal [1]. Traditionally, exploration of residency programs occurs during visiting electives where SMS travel across Canada to complete a series of two-week-long rotations at hospitals outside of their home institution [2,3]. SMS can also interview in-person with prospective residency programs and attend program informational sessions and socials [2,4]. Visiting electives and in-person interviews are therefore a key component of the CaRMS process, as they provide applicants with the opportunity to interact with staff and residents, understand the institutional culture, explore the city, and ultimately assess their “fit” with different programs [5]. However, in March 2020, the Association of Faculties of Medicine of Canada (AFMC) suspended visiting electives and in-person interviews indefinitely to limit the spread of COVID-19 [3,6]. This forced residency programs and applicants to find alternate avenues to share CaRMS-related information with one another.

Since the COVID-19 pandemic, the number of Canadian social media users increased from 27.66 million in 2019 to 35.81 million in 2023 [7]. Specifically, there has been increased use of platforms such as Instagram and TikTok by the healthcare community [8,9]. Twitter, a microblogging application that allows users to share short messages known as “tweets” [10], is cited as the most popular form of social media used by the medical community [8,9]. Throughout the pandemic, the number of global Twitter users increased 12-fold [11], as breaking COVID-related news was often shared first on Twitter [12]. Tweets can be tagged with a hashtag to allow users to easily filter for posts with related content [13]. Many healthcare-related hashtags (i.e., #COVID, #Surgery) are used by healthcare professionals on Twitter to share emerging research, circulate information, and connect with colleagues [8,9,12,14].

Since the suspension of visiting electives and in-person interviews, residency programs have increasingly relied on social media to advertise themselves [8,15]. Due to its accessibility and wide reach, Twitter has become a useful tool for residency programs to share real-time updates with CaRMS applicants regarding virtual events and deadlines [15-17]. To date, no study has analyzed whether the content of CaRMS-related tweets and user demographics has changed since the COVID-19 pandemic.

As such, the purpose of this study is to assess the content of CaRMS-related tweets from three cycles prior to and during the COVID-19 pandemic. Specifically, we aim to provide a better understanding of how educators and applicants are utilizing this social media platform to share CaRMS-related information and provide universities with information regarding their uptake of Twitter relative to other Canadian institutions.

## Materials and methods

Ethical statement

This study did not require ethics approval as data was collected from publicly available documents and online sources.

Study design

This study analyzed the content of CaRMS-related tweets and the demographics of the tweet creators prior to and during the COVID-19 pandemic. This study was exploratory in nature, given that it is the first study known to analyze the use of Twitter for sharing information related to the CaRMS process.

Procedure

**Figure 1:**
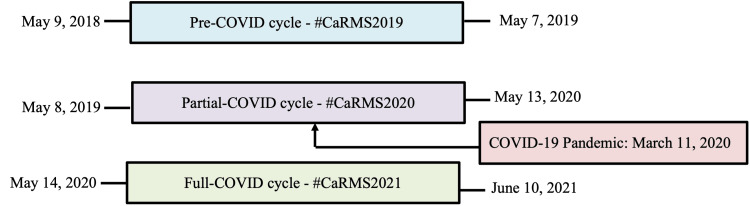
Timeline of three CaRMS cycles prior to and during the COVID-19 pandemic CaRMS: Canadian Resident Matching Services, COVID-19: coronavirus disease 2019

Three CaRMS cycles of interest and their relevant hashtag were defined: (1) pre-COVID, (2) partial-COVID, and (3) full-COVID cycles (Figure 1). The partial-COVID cycle corresponds to the 2019-2020 CaRMS year, where only the latter half of the cycle was impacted by the COVID-19 pandemic. The analytics tool Vicinitas was used to extract all tweets published within the predefined date range with the hashtags of interest. Tweets from all cycles were retrospectively collected from Vicinitas on June 18, 2021. Only primary tweets, defined as original posts made by a Twitter user, were eligible for inclusion. Retweets (i.e., reposting of an original tweet) or replies to original tweets were excluded. Basic tweet information including type (i.e., primary tweet, retweet, reply) and content of the tweet, date and time of publication, language, and username of the tweet creator was extracted. Vicinitas automatically extracts virality information, which details the number of times each tweet was favorited, retweeted, and replied to; however, it was not utilized in the final analysis. Using the date and time of publication extracted by Vicinitas, all included tweets were manually categorized into the appropriate CaRMS cycle by one study author (LV). The demographic information of the tweet creator including their profession (i.e., medical student, resident) and academic affiliation or the type of institution (i.e., residency program, national organization) and location were also manually extracted. If the user’s profession or academic affiliation could not be clearly identified in the tweet or user profile, external sources such as provincial regulatory college databases (i.e., College of Physicians and Surgeons of Ontario) and institutional websites were consulted.

Data analysis

Descriptive statistics were used to describe the demographic information of tweet creators. Categorical variables were summarized using frequencies and percentages. One study author (LV) deductively analyzed eligible tweets into one of the following nine codes: (1) CaRMS-related experience, (2) event advertisement, (3) information related to CaRMS, (4) negative match result, (5) positive match result, (6) program promotion, (7) reflection about CaRMS, (8) supportive message, or (9) other (i.e., physician sharing their match story). Tweets that could be categorized into multiple codes were coded in consultation with a second author (KA). The coding process was iterative and comparative, as sample tweets were presented to all study authors to ensure consistent interpretation of the codes and to identify overarching themes.

## Results

This study examined a total of 1,843 tweets published over three CaRMS cycles. Tweet characteristics are displayed in Table 1. Overall, 603 tweets (32.7%) were published during the pre-COVID cycle, 472 tweets (25.6%) in the partial-COVID cycle, and 768 tweets (41.7%) in the full-COVID cycle. Across all three cycles, the majority of tweets were written in English (97.4%) and posted by medical students (29.5%) and residents (16.4%) who were most often affiliated with the University of Toronto (11.6%) or McMaster University (11%). Tweet creators were located in 16 different countries; however, 94.3% of tweets originated from Canada.

**Table 1:** Tweet characteristics from three Canadian residency match cycles

Tweet characteristics	Pre-COVID (number (%))	Partial-COVID (number (%))	Full-COVID (number (%))
Total number of tweets	603 (100)	472 (100)	768 (100)
Tweet creators			
Academic institution	31 (5.1)	38 (8.1)	74 (9.6)
Family/friends	4 (0.7)	4 (0.8)	8 (1)
Medical school	10 (1.7)	13 (2.8)	43 (5.6)
Medical student	137 (22.7)	166 (35.2)	236 (30.7)
Organization (i.e., College of Family Physicians of Canada)	174 (28.9)	25 (5.3)	53 (6.9)
Program director	20 (3.3)	11 (2.3)	38 (4.9)
Residency program	20 (3.3)	20 (4.2)	96 (12.5)
Resident/fellow	85 (14.1)	90 (19.1)	122 (15.9)
Staff physician	85 (14.1)	79 (16.7)	73 (9.5)
Other (i.e., general social media user)	37 (6.1)	26 (5.5)	25 (3.3)
Language			
English	576 (95.5)	460 (97.5)	759 (98.8)
French	27 (4.5)	12 (2.5)	9 (1.2)
Location			
Dalhousie University	9 (1.5)	13 (2.8)	27 (3.5)
Memorial University of Newfoundland	2 (0.3)	46 (9.7)	49 (6.4)
McGill University	4 (0.7)	3 (0.6)	29 (3.8)
University of Ottawa	37 (6.1)	71 (15)	46 (6)
Queen’s University	33 (5.5)	20 (4.2)	28 (3.6)
University of Toronto	76 (12.6)	60 (12.7)	77 (10)
McMaster University	74 (12.3)	45 (9.5)	84 (10.9)
Western University	28 (4.6)	41 (8.7)	24 (3.1)
Northern Ontario School of Medicine	5 (0.8)	5 (1.1)	48 (6.3)
University of Manitoba	24 (4)	29 (6.1)	21 (2.7)
University of Saskatchewan	3 (0.5)	8 (1.7)	24 (3.1)
University of Calgary	9 (1.5)	11 (2.3)	60 (7.8)
University of Alberta	29 (4.8)	16 (3.4)	55 (7.2)
University of British Columbia	34 (5.6)	32 (6.8)	69 (9)
American university	1 (0.2)	4 (0.8)	4 (0.5)
International university	7 (1.2)	6 (1.3)	29 (3.8)

**Figure 2:**
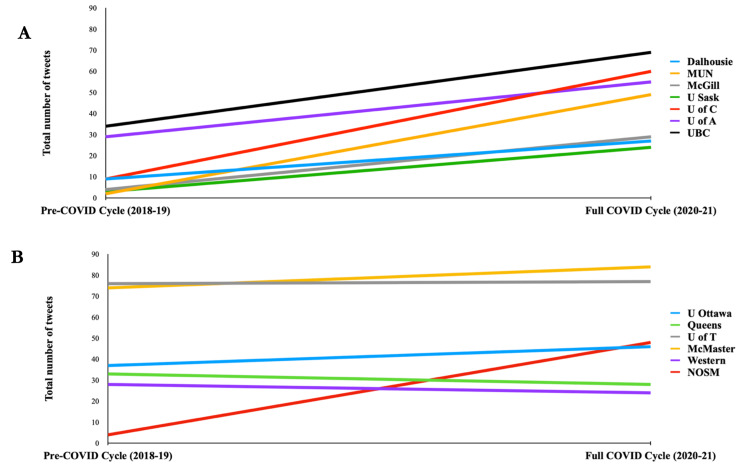
Trend in total published tweets based on academic affiliation over three CaRMS cycles: (A) total tweets from creators affiliated with institutions outside of Ontario before and during COVID-19 and (B) total tweets from creators affiliated with Ontario institutions before and during COVID-19 CaRMS: Canadian Resident Matching Services, COVID-19: coronavirus disease 2019, Dalhousie: Dalhousie University, MUN: Memorial University Newfoundland, McGill: McGill University, U Sask: University of Saskatchewan, U of C: University of Calgary, U of A: University of Alberta, UBC: University of British Columbia, U Ottawa: University of Ottawa, Queens: Queen’s University, U of T: University of Toronto, McMaster: McMaster University, Western: Western University, NOSM: Northern Ontario School of Medicine

The total number of tweets from each cycle categorized by academic affiliation is presented in Figure 2. Figure 2A demonstrates tweets created by creators affiliated with institutions outside of Ontario. Figure 2B depicts the total tweets from creators affiliated with institutions outside of Ontario. There was an overall increase in the total number of tweets from the pre-COVID cycle to the full-COVID cycle from all institutions outside of Ontario. Except for the Northern Ontario School of Medicine (NOSM), there was no notable increase or decrease in total tweets from Ontario institutions. The institutions with the greatest increase in total tweets between the first and third cycles were Memorial University Newfoundland (MUN), NOSM, and the University of Saskatchewan (U Sask), which saw a 25-, 10-, and eightfold increase, respectively.

**Figure 3:**
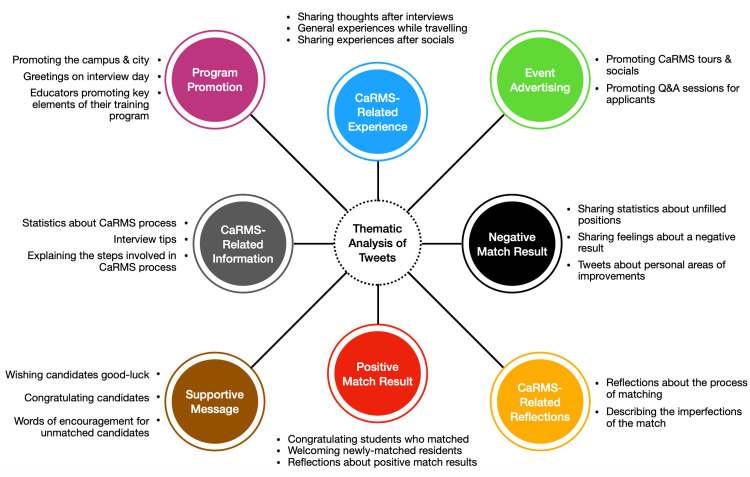
Summary of themes of all published CaRMS-related tweets CaRMS: Canadian Resident Matching Services

**Figure 4:**
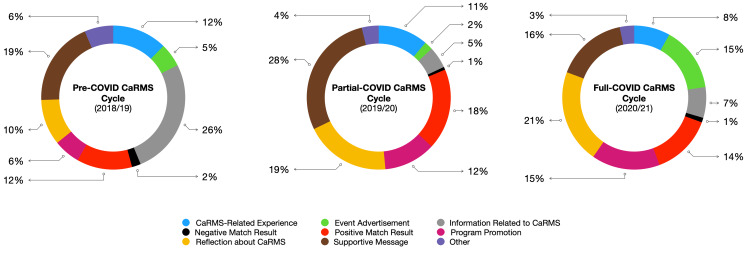
Thematic classification of tweets from three CaRMS cycles between 2018 and 2021 CaRMS: Canadian Resident Matching Services

Across all cycles, supportive messages (29.1%), reflections about CaRMS (24.7%), and positive match results (20.8%) were the most common types of tweets. Sample tweets from each category across all three cycles are displayed in Table 2 and summarized graphically in Figure 3. The number of tweets coded in each of the nine categories across all three cycles is displayed in Figure 4. Four of the nine most coded categories are described narratively below. The number of tweets from these categories was either consistently high across all cycles (i.e., supportive message) or changed considerably between cycles (i.e., event advertisement, program promotion, reflection about CaRMS).

**Table 2:** Sample CaRMS-related tweets across three cycles prior to and during the COVID-19 pandemic CaRMS: Canadian Resident Matching Services, COVID-19: coronavirus disease 2019

Category	Pre-COVID (2018-2019)	Partial-COVID (2019-2020)	Full-COVID (2020-2021)
CaRMS-related experience	First #CaRMS2019 interview: Spill water all over my pants. Immediately after getting called for my interview. Owned it. Had to. Interviewer replies, “at least it’s not coffee?” Hopefully uphill from here... (Or downhill? As a skier I genuinely get this expression confused.) - Medical Student, January 2019	Fourth-year medical students are getting @CaRMS_CA Match Day T-shirts to write where they’ll be doing their residencies. #CaRMSMatch #carms2020 - Academic Institution, March 2020	An absolute pleasure interviewing so many phenomenal applicants this weekend. The future of our speciality has never been brighter. Best of luck to everyone in the match! #carms2021 @CanUrolAssoc - Academic Institution, March 2021
Event advertisement	We are so excited to host all the #CaRMS2019 Candidates @MacEmerg tonight! We’ve booked a really cool venue and are excited to show you the best of the new Hamilton! #HamOnt - Academic Institution, January 2019	To any med students interested in hearing more about Manitoba’s Emergency Medicine Residency Program, we will be hosting our first Q&A zoom session on August 11 at 7pm Central Time. Msg us on Twitter or Instagram for the zoom link! We are so excited to meet everyone! #carms2020 - Residency Program, July 2020	Interested in more information about our residency program? Our first of 2 virtual information and Q&A sessions is on December 17! Open to all @CaRMS_CA 2021 applicants. Check out the link for more info! #carms2021 #physiatry #PMR @USASK_PGME (link inserted here) - Academic Institution, December 2020
Information related to CaRMS	We know @CaRMS_CA interviews can be a stressful experience for medical students - it’s why we’ve held mock interview training over the past month for more than 1000 students. Check out the video below to see how it went. #CaRMS2019 #MedEd - Organization, January 2019	Great new resource for year 4 MD students gearing up for #CaRMS2020, courtesy of @CFMSFEMC! #MedStudentTwitter - Medical School, October 2020	As the interviewee I'm biased, but if you're going through CaRMS soon (and particularly if you're applying to EM) I think this is worth a listen! Lots of great discussion on what is shaping up to be an exciting @WeAreCanadiEM podcast series. #CaRMS2021 - Medical Student, October 2020
Negative match result	Out of only 7 Vascular surgery CMG spots, 2 went unmatched! We were the only surgical specialty to go unmatched. How can we do better next year?? The future of our specialty depends on attracting the best and brightest to our dynamic specialty! #CaRMS2019 - Resident, February 2019	Some very scary stats coming out of our province after #carms2020 with nearly half of Family Med residency seats going unfilled. Though I personally don’t plan to do family I think there are many positives that aren’t being sold enough to students. - Medical Student, March 2020	Unmatched… again. Pretty crushed, but I know I put in the best application I possibly could have. Grateful for the opportunities I have gotten thus far, now on to new adventures. #carms2021 - Medical Student, April 2021
Positive match result	A dream come true! So happy to have matched to Ottawa Physiatry! #CaRMS2019 - Medical Student, February 2019	With the completion of #carms2020, we are thrilled to welcome six medical students into our Neurology and Neurosurgery residency programs this July. @SchulichMedDent #Schulichadvantage - Academic Institution, March 2020	We are thrilled to have matched all of our residency positions in each track! @UBC_Psychiatry warmly welcomes the outstanding new residents who will be joining our postgraduate training program ! #carms2021 - Academic Institution, April 2021
Program promotion	Monday night hangs with the residents of #OUPath. We interviewed an incredible group of applicants last week for #CaRMS2019 - I’d just like to remind you all that our program shows a demonstrated commitment to resident #wellness and offers a generous fund to support it! (image) - Resident, January 2019	Excited to host candidates at @QueensGenSurg for their @CaRMS_CA interviews in the new year! Here’s some info about our program, people, and city as you plan your trips here! @CAGS_Residents @CAGS_ACCG @CFMSFEMC @AFMC_e @CUSEC_Surgery #residency #carms2020 #generalsurgery (link to more information) - Resident, January 2020	We have adapted many ways of working these last 9 months and #CaRMS2021 info to help our future residents learn about us is the most recent change. Check out our YouTube channel #BrighterWorld @MacHealthSci (link to YouTube)” - Academic Institution, November 2020
Reflection about CaRMS	Perspective re: @CaRMS_CA matching process. What other professional degrees go to so much expense & time to set-up students for the next phase of training/career? Graduate students study for many years w no guarantee of next steps or high-payed prestigious career. #CaRMS2019 - Other, February 2019	After 4 days of helping with CaRMS interviews at @UofODFM, I have newfound appreciation for this wonderful department and the people in it. So lucky to be training here. + the future of family medicine is looking so bright! Thanks to all the #CaRMS2020 applicants for joining us! - Resident, January 2020	Thanks to a great team MedIT to our PA Jenni, #carms2021 is in the books! It was great meeting you all. The real tragedy is many of you will be ranking locations you may have never been. After all you’ve been through this past year, it’s a little unfair. - Program Director, March 2021
Supportive message	Nous souhaitons la meilleure des chances aux étudiants en médecine qui commencent les entrevues du #CaRMS2019. Voyagez en toute sécurité! (image) - Other, January 2019	Congratulations to all the medical students who matched yesterday in #CaRMS2020. 1.You're great no matter what the outcome. 2. You will learn a ton and have fun wherever you ended up. 3. You're great no matter what the outcome. - Resident, March 2020	Thinking of all my friends at @uOttawaMed (and elsewhere) who are starting #carms2021 interviews today. It's been a longer year than normal to get here... hold your heads high, be yourselves, and remember to unmute your mic 😉. You guys can do ANYTHING. - Resident, March 2021

Event advertisement

Within the pre-COVID cycle, 32 (5.3%) tweets were coded as event advertisements. Most tweets from this cycle advertised in-person socials held by the residency program. Within the partial-COVID cycle, nine (1.9%) tweets were coded as event advertisements and highlighted both in-person and virtual events. Within the full-COVID cycle, 112 (14.6%) tweets were coded as event advertisements, and all advertised online events held by the residency program such as informational sessions.

Program promotion

Within the pre-COVID cycle, 36 (6%) tweets were coded as program promotion and most commonly written to welcome students to in-person interviews and advertise redeeming qualities about the program. Within the partial-COVID cycle, 55 (11.7%) tweets were classified as program promotion. Similarly, tweets from this cycle advertised in-person interviews and promoted particularities of the city where the program was located. Within the full-COVID cycle, 118 (15.4%) tweets were coded as program promotion. Tweets from this cycle included links to the institution’s website and YouTube videos that highlighted supplemental program information.

Supportive message

Within the pre-COVID cycle, 115 (19.1%) tweets were coded as supportive messages and often displayed words of encouragement to support the SMS as they traveled across the country for CaRMS interviews. Within the partial-COVID cycle, 134 (28.4%) tweets were coded as supportive messages. Several tweets from this cycle congratulated students on matching to a program and wished luck to those awaiting results. Within the full-COVID cycle, 123 (16%) tweets were coded as supportive messages and encouraged applicants to persevere despite COVID-related obstacles that impacted the CaRMS process.

Reflection about CaRMS

Within the pre-COVID cycle, 62 (10.3%) tweets were coded as a CaRMS reflection. These tweets were from applicants who shared their experiences traveling across the country and interviewing with programs. A minority of tweets described individuals’ perspectives about the imperfections of the general CaRMS application process (i.e., faults of the matching algorithm, amount of time spent traveling). Within the partial-COVID cycle, 123 (19.3%) tweets were coded as a CaRMS reflection. Tweets from this cycle were similar to those pre-COVID. Within the full-COVID cycle, 162 (21.1%) tweets were coded as a CaRMS reflection. A large proportion of these tweets from both applicants and medical professionals (i.e., residents, physicians) mentioned the impact that COVID had on the application process. Tweet creators highlighted the benefits of having virtual events (i.e., better for the environment, convenience) and the disadvantages of this new format (i.e., inability to visit the school, lack of face-to-face interactions).

## Discussion

This study examined 1,843 tweets published across three CaRMS cycles prior to and during the COVID-19 pandemic to evaluate Twitter’s utility as a platform to share CaRMS-related information. To date, no study has analyzed the content of CaRMS-related tweets and the demographics of tweet creators. Previous research has evaluated the use of social media over the course of the pandemic by specialty-specific programs (i.e., general surgery, anesthesiology) and found that residency programs had increased use of social media in the years following the COVID-19 pandemic [15-17]. While the overall increase in total tweets noted in this study is in keeping with the literature, our data differ slightly as a transient decrease in total tweets between the pre-COVID cycle (2018-2019) and the partial-COVID cycle (2019-2020) from 608 to 472 was noted. We hypothesize that this unexpected decrease may be because medical professionals had a sudden increase in workload when COVID-19 was first declared a global pandemic [18,19], which resulted in less time spent using social media during 2020.

When examining the demographics of tweet creators, Twitter saw an increase in the number of tweets contributed by medical schools (fourfold), residency programs (fourfold), medical students (twofold), and program directors (twofold). These findings are in line with the literature [15-17], reporting increased use of social media during the pandemic by individuals directly involved with CaRMS applications. Interestingly, tweets created by organizations (i.e., College of Family Physicians of Canada) saw a threefold decrease between the pre-COVID and full-COVID cycles. This may be because some organizations narrowed their social media focus during the pandemic to convey information that is more closely aligned with issues directly related to COVID-19. For example, @CFPC_e shifted their focus to promote COVID-related content, such as mask-wearing and how to avoid burnout. A minority of organizations continued to tweet about CaRMS-related topics but excluded the CaRMS hashtag and therefore were not captured in our data.

There was an incidental geographic trend noted. Institutions located in cities with a population of less than 250,000, namely, Memorial University Newfoundland (MUN), NOSM, and University of Saskatchewan (U Sask), saw the greatest increase in total tweets between the pre- and full-COVID cycles. Although the lack of visiting electives and in-person interviews led to decreased visibility of all programs, our data suggest that institutions with the greatest need for self-promotion through social media were smaller institutions located in relatively less populated cities [20,21]. These geographic trends should be studied following the reinstatement of visiting electives to see if they persist. With the exception of NOSM, there was no overall trend in total tweets from creators affiliated with Ontario institutions. This may be due to the high use of social media pre-pandemic or no increased need for self-promotion during the COVID-19 pandemic.

The qualitative analysis demonstrated an increase in the number of tweets coded as “program promotions” and “event advertisements” from 6% and 5.3% during the pre-COVID cycle to 15.4% and 14.6% during the full-COVID cycle, respectively. The content within the tweets also evolved throughout the pandemic to include links to institutional websites, YouTube videos, and sign-up pages for virtual events. Interestingly, the number of tweets coded as “supportive tweets” overall decreased over the three cycles. While the reason is unclear, it is well documented that healthcare workers are reporting increased stress due to the emotional and physical toll associated with providing healthcare services in Canada [22]. Given that the full-COVID cycle took place during the second year of the pandemic, it is possible that an element of compassion fatigue carried over to social media and contributed to the decrease of supportive tweets toward SMS progressing through CaRMS [23,24]. Alternatively, much of social media’s support was directed to frontline workers; thus, it is possible that SMS were simply overlooked as a group in need of support [25].

Given that virtual CaRMS interviews have important financial and environmental benefits [26], they are likely to persist beyond the COVID-19 pandemic. As such, this provides an ongoing utility for tweets to convey important program-specific information to applicants who invariably cannot complete visiting electives at all institutions across the country. While this study provides useful insight into the utility of Twitter as a medium to share CaRMS-related information, our study is not without limitations. First, this study only examined the content of tweets with the following hashtags: #CaRMS2019, #CaRMS2020, and #CaRMS2021. Thus, it is possible that CaRMS-related tweets were not captured in our data because they did not contain one of these hashtags. However, given that a total of 1,843 tweets were examined in this study, our data likely represent overall trends throughout the COVID-19 pandemic. Second, this study does not examine the number of Twitter users who interacted with each tweet. Future studies may consider assessing how the number of individuals interacting with CaRMS-tagged tweets via replies or retweets has changed since the pandemic. Future studies may consider analyzing the use of Twitter following the reinstatement of visiting electives in the 2023-2024 CaRMS cycle to assess if these numerical and geographic trends prevail. Analyzing tweets over several additional cycles may help to establish long-term trends that may differ from those noted in this study, specifically analyzing the number of tweets by academic affiliation to identify changes in the geographical trends and in the common types of tweets (i.e., supportive tweets, event advertisements). Third, a qualitative study may be undertaken to evaluate how often CaRMS applicants interact with the CaRMS-related tweets identified in this study and whether applicants consider the information shared during the application process. Finally, determining if the residency programs with increased Twitter use also had increased interest in their program as measured by the total number of CaRMS applications may be valuable.

## Conclusions

This study examines Twitter as a platform to share CaRMS-related information prior to and during the COVID-19 pandemic. Overall, the number of tweets published by medical students, residents, and residency programs increased and were primarily created to promote programs and advertise CaRMS events. Institutions located in relatively rural locations had the greatest increase in tweets. Although visiting electives will be reinstated, CaRMS interviews will likely remain virtual given their financial and environmental benefits. This highlights the ongoing need for Twitter to convey important program-specific information to CaRMS applicants.
